# Designing Organic Spin-Gapless Semiconductors via Molecular Adsorption on C_4_N_3_ Monolayer

**DOI:** 10.3390/molecules29133138

**Published:** 2024-07-01

**Authors:** Dongqiu Zhao, Xiao Tang, Wanyan Xing, Yixin Zhang, Xueying Gao, Mengrui Zhang, Zhengao Xie, Xunwang Yan, Lin Ju

**Affiliations:** 1 School of Physics and Electric Engineering, Anyang Normal University, Anyang 455000, China; dqzhao@aynu.edu.cn (D.Z.); 221101039@stu.aynu.edu.cn (W.X.); 221101089@stu.aynu.edu.cn (Y.Z.); 221101050@stu.aynu.edu.cn (X.G.); 221101042@stu.aynu.edu.cn (M.Z.); 2 Institute of Materials Physics and Chemistry, College of Science, Nanjing Forestry University, Nanjing 210037, China; xiaotang@njfu.edu.cn; 3 College of Physics and Engineering, Qufu Normal University, Qufu 273165, China; xiezhengao163@163.com

**Keywords:** spin-gapless semiconductor, spintronics, density functional theory calculations, C_4_N_3_

## Abstract

Spin-gapless semiconductor (SGS), a class of zero-gap materials with fully spin-polarized electrons and holes, offers significant potential for high-speed, low-energy consumption applications in spintronics, electronics, and optoelectronics. Our first-principles calculations revealed that the Pca21 C_4_N_3_ monolayer exhibits a ferromagnetic ground state. Its band structure displays SGS-like characteristics, with the energy gap between the valence and conduction bands near the Fermi level in the spin-down channel much smaller than the one in the other spin channel. To enhance its SGS properties, we introduced electrons into the Pca21 C_4_N_3_ monolayer by adsorbing the CO gas molecule on its surface. Stable gas adsorption (CO@C_4_N_3_) effectively narrowed the band gap in the spin-down channel without changing the band gap in the spin-up channel obviously. Moreover, injecting holes into the CO@C_4_N_3_ system could increase the net magnetic moments and induce an SGS-to-metallic phase transition, while injecting electrons into the CO@C_4_N_3_ system is able to lower the net magnetic moments and cause an SGS-to-half-metallic phase transition. Our findings not only underscore a new promising material for practical metal-free spintronics applications but also illustrate a viable pathway for designing SGSs.

## 1. Introduction

Spintronics aims to utilize electron spin in conjunction with an electrical charge for the development of logic and memory devices, revolutionizing information processing [1]. An essential challenge that fuels innovation in this domain is achieving the generation of 100% spin-polarized currents at the Fermi level. In addition to half-metals [2,3], spin-gapless semiconductors (SGSs) also meet the demand for materials with unique electronic properties. SGSs are characterized by having a zero gap in one spin channel and a finite gap in the other. The electronic structures of SGSs are highly sensitive to external effects since electrons can transition from occupied states to empty ones without requiring any threshold energy [4]. Both the excited electrons and holes in SGSs are fully spin-polarized, making them highly promising for spintronics applications like spin-current generators and semiconductor injectors. Remarkably, numerous SGSs exhibit inverted band structures and significant bulk band gaps due to spin–orbit coupling (SOC) [5], thereby acting as topological insulators [6,7].

SGSs can be divided into two types according to whether their band structures near the Fermi level exhibit linear or parabolic energy–momentum dispersion [8]. In SGSs with linear dispersion, the charge carriers (electrons and holes) act as spin-polarized massless Dirac fermions. These characteristics have been anticipated in triangular ferrimagnets [9] and two-dimensional (2D) metal–organic frameworks [10,11]. Heusler compounds, including Co-doped PbPdO_2_ and Mn_2_CoAl [8,12,13,14], have been shown to possess parabolic energy–momentum dispersion. Additionally, BN sheets doped with transition metal (TM) and metal-free BN nanoribbons containing vacancies are also predicted to exhibit parabolic dispersive SGS characteristics [15,16]. While graphene naturally has a linear dispersion relation, it is spin-degenerated. However, cutting graphene into strips along specific directions can create one-dimensional SGS [17]. Notably, 2D SGSs consistently include TM atoms, which play a role in electron spin polarization. So far, there has been a scarcity of metal-free SGSs. The benefit of metal-free SGSs lies in their extended diffusion lengths and prolonged coherence times, resulting from weak spin–orbital and hyperfine interactions. These features offer optimal conditions for coherent spin manipulation.

Recently, interest in 2D graphitic carbon nitrides has surged due to their potential applications in photocatalysis [18,19] and hydrogen storage [20,21]. These two primary building blocks of 2D-graphitic carbon nitride materials are s-triazine (C_3_N_3_) and tri-s-triazine (C_6_N_7_) [22]. Recent theoretical studies have revealed that g-C_6_N_6_ exhibits topologically nontrivial electronic states and can be converted into a topological insulator through doping [23]. Additionally, the hybrid honeycomb lattice composed of C_7_N_6_ and C_3_N_3_ units has been identified as an SGS with parabolic energy–momentum dispersion relations near the Fermi level [24]. These unique properties suggest that graphitic carbon nitride materials hold promise for the exploration of 2D metal-free SGSs. In a recent breakthrough, a new type of stable graphitic carbon nitride material (Pca21 C_4_N_3_ monolayer) has been predicted by using a random method based on group and graph theory (RG2) [25]. Our first-principles calculations revealed that the Pca21 C_4_N_3_ monolayer exhibits a ferromagnetic ground state. Its band structure displays SGS-like characteristics. The energy gap between the valence and conduction bands near the Fermi level for the spin-down channel is only 0.36 eV, while the other spin channel has a comparatively larger band gap of 2.24 eV. To enhance its SGS properties, we introduced electrons into the Pca21 C_4_N_3_ monolayer by adsorbing CO gas molecule on its surface. Stable CO gas adsorption effectively narrowed the band gap in the spin-down channel without changing the band gap for the spin-up channel obviously. Additionally, the CO@C_4_N_3_ system’s net magnetic moment and electronic phase could be tuned by injecting holes/electrons.

## 2. Results and Discussion

### 2.1. Geometric Structure and Stability of Pure Pca21 C_4_N_3_ Monolayer

**Figure 1:**
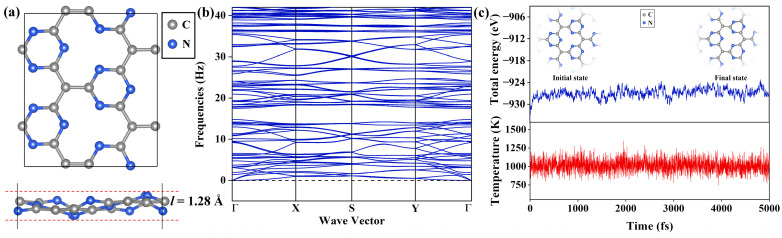
(**a**) Top and side views of the Pca21 C_4_N_3_ monolayer. (**b**) Phonon spectrum of the Pca21 C_4_N_3_ monolayer. (**c**) The AIMD simulations of the 4 × 4 C_4_N_3_ supercell at 1000 K, which show the total energy (upper) and temperature (upper) fluctuations with a time-step of 1 fs for 5 ps.

The C_4_N_3_ monolayer is essentially a graphitic C_3_N_4_ monolayer, in which some nitrogen atoms are replaced by carbon atoms. As shown in Figure 1a, the Pca21 C_4_N_3_ supercell exhibits a highly corrugated configuration, which has been confirmed as the ground state by the previous work [25], and its thickness *l* is 1.28 Å. The Pca21 C_4_N_3_ monolayer is composed of alternately arranged 12-membered macrocycles and 6-membered microcycles. Both the macrocycles and microcycles are constructed by two types of covalent bonds, i.e., C-C and C-N bonds. The C-C bond lengths range from 1.450 to 1.463 Å, with an average value of 1.455 Å, while the length of the C-N bond is between 1.340 and 1.359 Å, with an average value of 1.353 Å.

Then, we explore the dynamical and thermal stability of the Pca21 C_4_N_3_ monolayer in turn. The dynamic stability is checked through the phonon spectra analyses. As displayed in Figure 1b, there are no imaginary frequencies, ensuring dynamic stability. To further demonstrate the thermal stability of the Pca21 C_4_N_3_ monolayer, we built a 4 × 4 supercell containing 112 atoms and performed the spin-polarized ab initio molecular dynamics (AIMD) simulations with a 1000 K Nosé–Hoover thermostat. As illustrated in Figure 1c, the atomic configuration of the Pca21 configuration of C_4_N_3_ remained intact, exhibiting only minor deformations. The total energy oscillations were merely in a very narrow range. Both of them confirm that the Pca21 C_4_N_3_ monolayer is dynamically and thermally stable.

### 2.2. Electronic Structure and Magnetic Properties of Pure Pca21 C_4_N_3_ Monolayer

**Figure 2:**
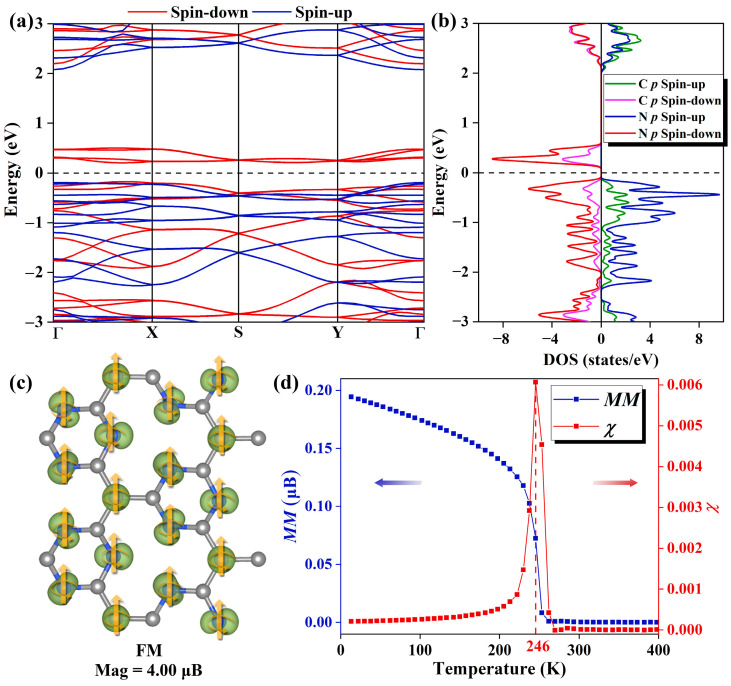
(**a**) The spin-resolved band structure of the Pca21 C_4_N_3_ monolayer features the spin-up bands in blue and the spin-down bands in red. The Fermi level, indicated by the black dashed line, is set to 0 eV. (**b**) The spin-resolved PDOS plots of the C and N 2*p* orbitals of Pca21 C_4_N_3_ monolayer. In the spin-up channel, the N 2*p* and C 2*p* orbitals are represented by blue and green lines, respectively, while in the spin-down channel, the N 2*p* and C 2*p* orbitals are indicated by purple and red lines, respectively. (**c**) The 3D isosurfaces (the iso-value is 0.01 e/au) of net magnetization density (spin-up minus spin-down) of the Pca21 C_4_N_3_ monolayer in FM state. (**d**) The Heisenberg model magnetization susceptibility *χ* and average magnetic moment *M* as a function of temperature.

As plotted in Figure 2a, the spin-resolved electronic band structure demonstrates that the Pca21 C_4_N_3_ monolayer shows an SGS-like performance. It has a small band gap (0.36 eV) between the conduction band minimum (CBM) and valence band maximum (VBM) in the spin-down channel, while the one in the spin-up channel is much larger (2.24 eV). Based on the partial density of states (PDOS) shown in Figure 2b, we find that in the spin-down channel, both the CBM and the VBM are primarily composed of N 2*p* orbitals. In the spin-up channel, the CBM is formed by N 2*p* and C 2*p* orbitals equally, while the VBM is mainly derived from N 2*p* orbitals. Moreover, the PDOS reveals a fully spin-polarized state with a magnetic moment of 4.00 µB. A detailed analysis of the spatial spin density distribution provides better insight into the distribution of magnetic moments. Figure 2c illustrates the spatial spin density distributions of the Pca21 C_4_N_3_ monolayer, indicating that the magnetic moments are primarily localized on the N atoms and the C_M_ atoms. C_M_ atoms are those C atoms that only bond to the C atoms, and the C atoms with no magnetic moments are labeled as C_N_ atoms (as shown in Figure S1). As mentioned before, the C_4_N_3_ monolayer could be regarded as the C-doped C_3_N_4_ monolayer. The C_M_ position is the position where the substitution occurs. Considering the C_3_N_4_ monolayer is non-magnetic, we think the magnetic moments of the N atoms are induced by the magnetic C_M_ atoms. In order to explore the effect of spin direction on the band structure, we carefully computed the band structure properties of the system with spin orientations along the x, y, and z directions, respectively. As shown in Figure S2, the spin orientation has a minimal impact on the band structure. These phenomena could be explained by the absence of heavy metal elements in the system, which results in negligible magnetic anisotropy.

Deriving from the premise that the magnetic moments on the N atoms are influenced by the magnetic moments of the C_M_ atoms, as plotted in Figure S3, we designed two magnetic sequences, i.e., ferromagnetic (FM) and antiferromagnetic (AFM) states. We determined the type of magnetic coupling in the Pca21 C_4_N_3_ monolayer according to the energy deference (∆*E* = *E*_AFM_ − *E*_FM_) between the total energy of the FM state and that of the AFM state. The positive calculated ∆*E* (0.28 eV) means that the Pca21 C_4_N_3_ monolayer is more stable in the FM state. The nearest-neighbor exchange coupling constant *J* [26] for the Pca21 C_4_N_3_ monolayer was calculated, as depicted in Figure S3. By calculating the relationship between the energy and the magnetic interaction in the lattice for both magnetic orders, the following equations can be obtained:

(1) $$\frac{1}{4}(E_{FM}-E_{NM})=3E_{F}$$

(2) $$\frac{1}{4}\left(E_{AFM}-E_{NM}\right)=E_{F}+2E_{F}$$

where *E*_FM_ is the total energy of the ferromagnetic state, *E*_AFM_ is the total energy of the co-linear antiferromagnetic state, and *E*_NM_ is the total energy of the non-magnetic state. *E*_F_ is the energy difference due to the ferromagnetic interaction between two C_M_ atoms, and *E*_A_ is the energy difference due to the antiferromagnetic interaction between two C_M_ atoms. Considering the periodic arrangement, the ferromagnetic order of a C_M_ atom around the six C_M_ atoms has ferromagnetic interactions with it, excluding the duplication between the two atoms; the coefficient is 3. The coefficient in the colinear antiferromagnetic order can be obtained in the same way. By joining the above two equations, we can obtain

(3) $$J=\frac{1}{16}\left(E_{FM}-E_{AFM}\right)=-17.9\;meV$$

Curie temperature (T_c_) is the critical temperature at which the ferromagnetic–paramagnetic phase transition occurs in a material and is a key parameter in the study of ferromagnetic materials. By Monte Carlo simulation [27], the T_c_ of Pca21 C_4_N_3_ monolayer is obtained to be 246 K, and the images of magnetization susceptibility *χ* and average magnetic moment *M* with temperature are shown in Figure 2d.

### 2.3. Magnetic Property of Pure Pca21 C_4_N_3_ Bilayer

We also explore the possible magnetic coupling in the Pca21 C_4_N_3_ bilayer. We first construct three stacking modes, which are AA, AB, and AC patterns. In the AA stacking pattern, the C and N atoms of the upper layer recombine with the corresponding C and N atoms of the lower layer. In the AB stacking pattern, the C and N atoms of the upper layer overlap with the N and C atoms of the lower layer, meaning the AB arrangement is shifted by one C-C bond distance relative to the AA stacking pattern. In the AC stacking pattern, the C and N atoms of the upper layer coincide with the C and N atoms of the lower layer, indicating that the AC stacking pattern is shifted by one C-N bond distance relative to the AB stacking pattern. As shown in Figure S4, after full relaxation, the interlayer spaces of AA, AB, and AC stacking patterns are 3.54, 3.71, and 3.70 Å, respectively. According to Equation (5), the calculated formation energy *E*_for_ for AA, AB, and AC stacking patterns are −13, −13, and −14 meV/Å^2^, respectively. These negative values of *E*_for_ ensure the energetical stability of these bilayers, respectively. Due to the most negative *E*_for_, the AC stacking pattern is considered the most stable stacking pattern. Additionally, the AIMD result promises the thermal stability of the bilayer with the AC stacking pattern (seeing Figure S5). Subsequently, as displayed in Figure S6, we also designed FM and AFM states for the bilayer model with the AC stacking pattern. The energy deference (∆*E* = *E*_AFM_ − *E*_FM_) is 0.14 eV. Thus, the Pca21 C_4_N_3_ bilayer in the FM state is more stable, the same as the results of the Pca21 C_4_N_3_ monolayer. Additionally, the size of the interlayer exchange coupling is calculated to be −0.9 meV. Compared to the intralayer magnetic interactions, its contribution to the energy is very small, and its effect on the calculation of the Curie temperature is negligible. Therefore, it can be determined that the Curie temperature of the bilayer C_4_N_3_ is essentially the same as that of the C_4_N_3_ monolayer. We further calculated the spin-polarized electronic band structure and total DOS (TDOS) of the pure Pca21 C_4_N_3_ bilayer with the AC stacking pattern. As shown in Figure S7, compared to the pure Pca21 C_4_N_3_ monolayer, the spin-down band gap is reduced from 0.36 to 0.24 eV, and the spin-up band gap is reduced from 2.24 to 2.13 eV. Since the reduction in the spin-down band gap is significantly greater than that in the spin-up band gap, the formation of the bilayer structure has effectively enhanced the SGS performance.

### 2.4. Improving SGS Property with Electron Injection through CO Adsorption

**Figure 3:**
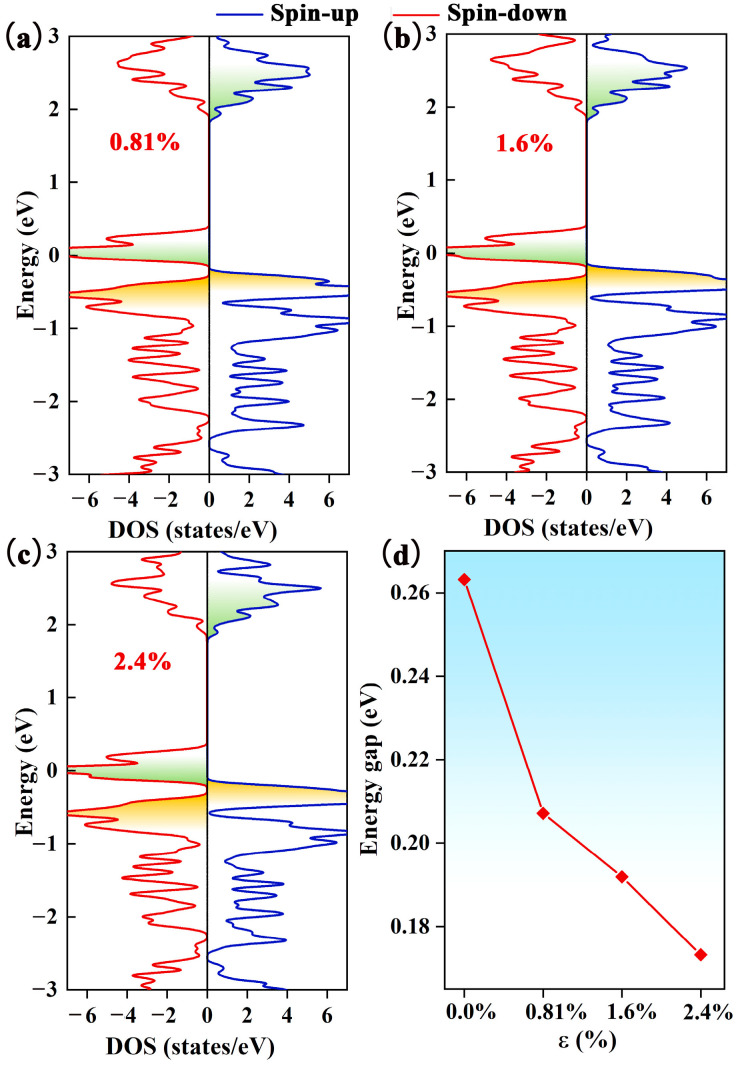
The DOS of the Pca21 C_4_N_3_ monolayer with the electron injection ratios of (**a**) 0.81%, (**b**) 1.6%, and (**c**) 2.4%. The blue line represents the spin-up states, while the red line indicates the spin-down states. (**d**) The relationship between the energy gap and electron injection ratio *ε*.

As mentioned earlier, the Pca21 C_4_N_3_ monolayer has an SGS-like electronic band structure. To enhance the SGS performance, we need to reduce the spin-down band gap by moving the CBM downward and/or shifting the VBM. To realize this operation, we increase the electron concentration of the Pca21 C_4_N_3_ monolayer. Based on the DOS calculation (as displayed in Figure 3a–c) of the Pca21 C_4_N_3_ monolayer with the introduction of electrons, we find that the spin-down band gap decreases with the increasing electron injection ratio (seeing Figure 3d). The spin-down band gap narrows from 0.26 to 0.17 eV when the extra electron concentration increases from 0 to 2.4%. In addition, the spin-up band gap remains almost unchanged. Therefore, the electron injection is able to effectively improve the SGS property.

**Figure 4:**
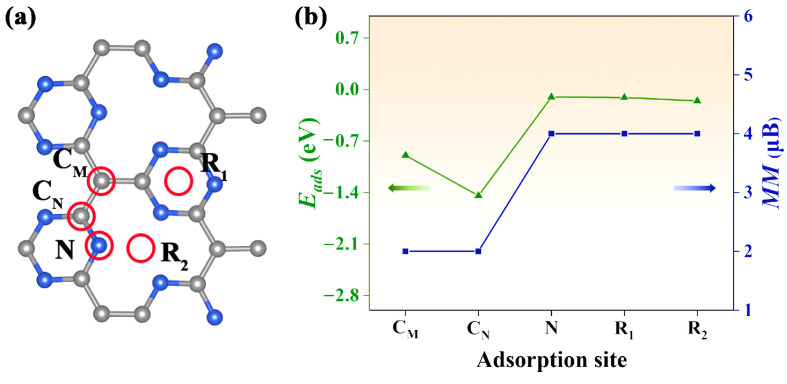
(**a**) The red circles indicate the adsorption sites considered in our study. The blue spheres are the nitrogen atoms, and the grey spheres are the carbon atoms. (**b**) The adsorption energy and net magnetic moment of the system with CO gas at different adsorption sites. The green line indicates the adsorption energy, while the blue line represents the magnitude of the magnetic moment.

An open question is how to realize electron doping in the Pca21 C_4_N_3_ monolayer. It is well known that gas adsorption on the surface of nanomaterial can cause a hole or electron injection, which normally results in a significant change in the electronic structure and magnetic properties [28]. In our study, the CO gas molecule was selected to be adsorbed on the Pca21 C_4_N_3_ monolayer. As shown in Figure 4a, we considered five adsorption sites: **C_M_**, the top site above the C_M_ atom; **C_N_**, the top site above the C_N_ atom; **N**, the top site above the N atom; **R1**, the top site above the center of the 6-membered microcycle; **R2**, the top site above the center of the 12-membered macrocycle. The CO molecule is initially vertical to the monolayer surface. According to Equation (6), we computed the adsorption energy *E*_ads_ for each adsorption configuration. By comparing these *E*_ads_ plotted in Figure 4b, we confirmed the most stable adsorption configuration (**C_M_** adsorption site) with the most negative *E*_ads_ (1.14 eV). Interestingly, we found that the net magnetic moment of the CO@C_4_N_3_ system could be tuned by the gas adsorption sites. The net magnetic moments (about 2 μB) of the CO@C_4_N_3_ systems with **C_M_** and **C_N_** adsorption sites are less than the ones (about 4 μB) of the CO@C_4_N_3_ systems with **N**, **R_1_**, and **R_2_** adsorption sites.

**Figure 5:**
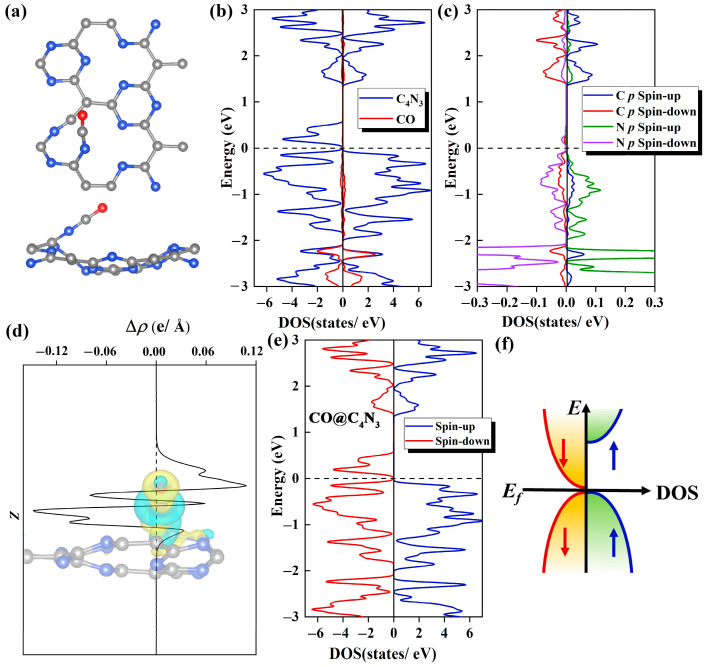
(**a**) The top (upper) and side (lower) views of the optimized structure for the Pca21 C_4_N_3_ monolayer with one CO molecule adsorbed on it. (**b**) The CO and C_4_N_3_ PDOS plots of the CO@C_4_N_3_ system. The C_4_N_3_ portion is denoted with a blue line, and the CO portion is denoted with a red line. (**c**) The PDOS plots of C 2*p* orbitals (denoted with red and blue lines for the spin-down and spin-up, respectively) from the adsorbed CO molecule and N 2*p* orbitals (denoted with purple and green lines for the spin-down and spin-up, respectively) from the N atom in the substrate, which bonded to the CO molecule. (**d**) Integrals of differential charge densities along the z direction for the CO@C_4_N_3_ system. The inset shows the differential charge density distributions, where yellow areas indicate electron accumulation and blue areas indicate electron depletion. The isosurface value is set to 5 × 10^−3^ e/Å^3^. (**e**) The TDOS plot for the CO@C_4_N_3_ system, with the blue line representing the spin-up states and the red line representing the spin-down states. (**f**) A diagram of the SGS DOS with a parabolic-type dispersion.

As shown in Figure 5a, in the most stable CO@C_4_N_3_ system, the C atom of CO faces downward, while the O atom faces upward. The C atom from the CO molecule is bonded with the N atom from the substrate. The N-C bond and the C-O bond are almost in a straight line, and the value of ∠NCO is 28°. Remarkably, the formation of a new C-N bond between the adsorbed gas and substrate makes an original C-N bond in the substrate break, producing an irregular fourteen-membered ring.

The work mechanism for this adsorption of CO gas molecule adsorbed on Pca21 C_4_N_3_ monolayer is meticulously investigated from the aspects of DOS, charge density difference (CDD), and Bader charge analysis. In the DOS diagram, we can observe that there is a significant electronic orbital hybridization between the CO gas molecule and substrate, which mainly focuses on the range from −1 to −3 eV (Figure 5b). This indicates a strong interaction between them, explaining the phenomenon that CO is tightly attached to the Pca21 C_4_N_3_ monolayer. Secondly, as shown in Figure 5c, the electronic orbital hybridization is mainly contributed by the coupling between the 2*p* orbitals of the C atoms from the CO molecule and the 2*p* orbitals of the N atoms from the substrate. The remarkable hybridization between the C and N atoms suggests the existence of a strong orbital interaction, leading to the formation of C-N chemical bonds. According to the CDD plot shown in Figure 5d, we find the substrate has some electron aggregation regions (yellow areas), which means some electrons have been injected into the Pca21 C_4_N_3_ monolayer after the CO adsorption. The Bader charge results also corroborate this point, revealing that the Pca21 C_4_N_3_ monolayer in the CO@C_4_N_3_ system has been doped with 0.8 *e* through CO adsorption.

In order to investigate whether CO adsorption on C_4_N_3_ can make the C_4_N_3_ band gap decrease, we calculated the TDOS of the CO@C_4_N_3_ system. Compared with the case of the pristine C_4_N_3_ monolayer, the spin-down band gap of the CO@C_4_N_3_ system (Figure 5e) is significantly reduced (almost 0 eV), while the large spin-up band gap remains essentially unchanged. Hence, the SGS property of the Pca21 C_4_N_3_ monolayer is enhanced with the CO adsorption. By comparing the TDOS of the CO@C_4_N_3_ system with that of an ideal parabolic-type SGS (as shown in Figure 5f), we believe that CO@C_4_N_3_ is a promising SGS.

Moreover, the thermal stability of the adsorption system needs to be checked for practical applications. Based on Equation (7), the recovery time of the C_4_N_3_ monolayer is calculated to be 1.67 × 10^11^ s at room temperature (300 K). The ultra-long desorption time promises a thermally stable adsorption configuration. Therefore, CO gas adsorption could effectively and stably enhance the SGS performance of the Pca21 C_4_N_3_ monolayer.

### 2.5. Adjusting the Magnetic Moment and Electrical Conductivity of CO@C_4_N_3_ through Electron/Hole Injection

**Figure 6:**
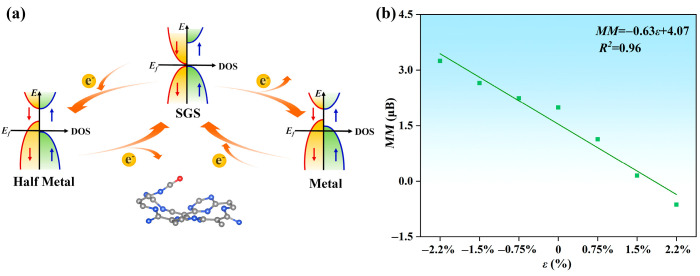
(**a**) Schematic diagram illustrating the mutual conversion between SGS, metallic, and half-metallic states in the CO@C_4_N_3_ system through the regulation of hole and electron injection. (**b**) The linear relationship between the injection rate *ε* of electrons and the net magnetic moment *MM* in the CO@C_4_N_3_ system. The negative value of the electron injection rate means the positive value of the hole injection rate. The linear relationship could be described as MM=−0.63ε+4.07, with the determination coefficient *R*^2^ of 0.96.

To further modulate the magnetic and electrical properties of the CO@C_4_N_3_ system, we injected different concentrations of electrons and holes into it. As shown in Figure S8a–c, as the injected hole concentration increases, the Fermi level traverses through the valence bands of both spin directions, reaching deeper energy levels within the valence band. The CO@C_4_N_3_ system transforms from the SGS state to the metallic (M) state, allowing electrons of both spin orientations to pass through it (as shown in Figure 6a). Moreover, the hole injection causes an increasing magnetic moment in the CO@C_4_N_3_ system (as displayed in Figure 6b). The total magnetic moments of the system are 2.24, 2.65, and 3.24 μB under the hole injection ratio of 0.75%, 1.5%, and 2.2%, respectively. Regarding the case of electron injection, the results are illustrated in Figure S8d–f. Along with the increasing injected electron concentration, the Fermi level traverses through the conduction band only in the spin-down direction, reaching deeper energy levels within that band. This transformation causes CO@C_4_N_3_ to shift from the SGS state to the half-metallic (HM) state while maintaining its 100% spin polarization property (as shown in Figure 6a). Furthermore, electron injection leads to a reduction in magnetic moments and even a reversal of spin orientation in the CO@C_4_N_3_ system (as displayed in Figure 6b). The total magnetic moments of the system are 1.13, 0.15, and −0.63 μB under electron injection ratios of 0.75%, 1.5%, and 2.2%, respectively. Hence, the SGS CO@C_4_N_3_ system exhibits a linearly charge-tunable magnetic moment, and when doped with holes (electrons), it becomes a stable metal (half-metal). This enables it to be of greater value in the spintronics apparatus.

## 3. Computation Details

The fundamental calculations were conducted within the framework of density functional theory (DFT), utilizing the DS-PAW package [29] and the Vienna ab initio Simulation Package (VASP code) [30]. The Perdew–Burke–Ernzerhof (PBE) form was used to treat the exchange-correlation function with the generalized gradient approximation (GGA) [31]. The projector-augmented wave (PAW) method was utilized to model the electron–ion interaction [29]. The computations employ a DFT+D3 method in Grimme’s scheme to account for the van der Waals (vdW) correction [32,33]. The vacuum space in the non-periodic direction exceeds 20 Å, ensuring no interaction between periodic images. During geometric optimizations, all atoms in the supercell were allowed to relax, with force convergence set at 0.05 eV/Å and total energy convergence at 10^−5^ eV. A 5 × 5 × 1 Monkorst–Pack k-point grid was applied to simulate the Brillouin zone interaction. The energy cutoff for plane-wave expansion of electron wavefunctions was set to 500 eV. Spin-polarization was considered in all the calculations. The phonon spectra were computed using a finite displacement method implemented in the Phonon code [34] alongside the VASP code. The Pca21 C_4_N_3_ monolayer in a ferromagnetic state was selected to obtain the phonon spectra [25].

In this paper, the Heisenberg model was used to describe the magnetic interactions of the system, and the Hamiltonian quantity was

(4) $$H=J\sum_{<i,j>}{\vec{S}}_{i}\cdot {\vec{S}}_{j}$$

where ${\vec{S}}_{i}$ and ${\vec{S}}_{j}$ denote the magnetic moments of the C_M_ sites, and <i,j> represents the nearest neighbor sites.

The stability of the Pca21 C_4_N_3_ bilayer was determined from the *E*_for_, which could be obtained as follows:

(5) $$E_{for}=(E_{bil}-2\times E_{mon})/S$$

where $E_{bil}$ and $E_{mon}$ represent the total energy of the Pca21 C_4_N_3_ bilayer and monolayer, respectively, while *S* denotes the area of the interface.

The adsorption energy $E_{ads}$ for a single CO molecule on Pca21 C_4_N_3_ monolayer is referred to as:

(6) $$E_{ads}=E_{total}-E_{CO}-E_{mon}$$

where $E_{total}$ and $E_{CO}$ are the total energy of the adsorption system and isolated CO molecule, respectively.

Based on the Van’t Hoff Arrhenius’ theory, the recovery time τ can be estimated by [35,36]:

(7) $$\tau =\omega^{-1}\exp\left(\frac{E^{*}}{K_{B}T}\right)$$

where *T*, K_B_, E*, and *ω* stand for the temperature, Boltzmann’s constant, desorption energy barrier, and attempt frequency. Here, E* is approximated as $E_{ads}$, while ω is set to 10^13^ s^−1^ [36].

## 4. Conclusions

SGS materials feature distinctive band structures where the valence and conduction bands meet at the Fermi level for one spin channel while the other spin channel exhibits a non-zero band gap. Based on the first-principles calculations, we find that the Pca21 C_4_N_3_ monolayer has a ferromagnetic ground state. Its band structure shows SGS-like characteristics, with the energy gap between the valence and conduction bands near the Fermi level for the spin-down channel being much smaller than that of the spin-up channel. Its dynamical and thermal stability have been confirmed by the phonon spectra analyses and AIMD simulations, respectively. Interestingly, stable CO gas adsorption can effectively narrow the band gap for the spin-down channel without significantly altering the band gap for the spin-up channel, thereby enhancing the SGS performance of the Pca21 C_4_N_3_ monolayer. Moreover, injecting holes into the CO@C_4_N_3_ system can induce a transition from SGS to a metallic phase, while injecting electrons is able to cause a transition from SGS to a half-metallic phase. Therefore, the SGS state bridges the metallic and the half-metallic states. Furthermore, the hole injection causes an increasing magnetic moment, while electron injection leads to a reduction in magnetic moment and even a reversal of spin orientation. Our comprehensive findings not only underscore a new, highly promising material suitable for practical applications in metal-free spintronics, offering significant advantages in terms of cost-effectiveness and environmental sustainability but also provide a detailed illustration of a viable and innovative pathway for designing SGSs, thereby paving the way for future advancements in this cutting-edge technology.

## Data Availability

Data are contained within the article and Supplementary Materials.

## Supplementary Materials

The following supporting information can be downloaded at: https://www.mdpi.com/article/10.3390/molecules29133138/s1, Figure S1: Atomic structure of C_4_N_3_ monolayer, yellow spheres are C atoms C_M_ with magnetic moments, silver spheres are C atoms C_N_ without magnetic moments, blue spheres are N atoms; Figure S2: The spin-resolved band structures of the Pca21 C_4_N_3_ monolayer with spin orientations along x, y, and z directions; Figure S3: Schematic diagram of ferromagnetic and co-linear antiferromagnetic order; Figure S4: The side and top views for AA, AB, and AC stacking partterns for Pca21 C_4_N_3_ bilayer; Figure S5: The AIMD simulations of the 2 × 2 C_4_N_3_ bilayer at 1000 K show the total energy and temperature fluctuations with a time-step of 1 fs for 5 ps; Figure S6: Schematic diagram of ferromagnetic and antiferromagnetic coupling of Pca21 C_4_N_3_ bilayer; Figure S7: The spin-resolved band structure and TDOS of the Pca21 C_4_N_3_ bilayer; Figure S8: DOS plots of the CO@C_4_N_3_ system with the hole injection ratio of 2.2%, 1.5%, and 0.75%, and the electron injection ratio of 2.2%, 1.5%, and 0.75%.
