# Supplementary material for: Designing Organic Spin-Gapless Semiconductors via Molecular Adsorption on C4N3 Monolayer
Source: Molecules. 2024 Jul 1;29(13):3138. doi: 10.3390/molecules29133138 (PMC11243529; doi:10.3390/molecules29133138)
Supplement: Supplementary file 1 [file molecules-29-03138-s001.zip › molecules-3074600-supplementary.pdf]

Supporting Information

Designing Organic Spin-Gapless Semiconductors via Molecular Adsorption on C_4N_3 Monolayer

Dongqiu Zhao [1], Xiao Tang [2], Wanyan Xing [1], Yixin Zhang [1], Xueying Gao [1], Mengrui Zhang [1], Zhengao Xie [3], Xun-Wang Yan [3,*], and Lin Ju [1,*]

[1]School of Physics and Electric Engineering, Anyang Normal University, Anyang, 455000, China

[2]College of Science, Institute of Materials Physics and Chemistry, Nanjing Forestry University, Nanjing 210037, China

[3]College of Physics and Engineering, Qufu Normal University, Qufu 273165, China

[*] **Corresponding authors**.

E-mail address: yanxunwang@163.com (X.Y.); julin@aynu.edu.cn (L.J.)

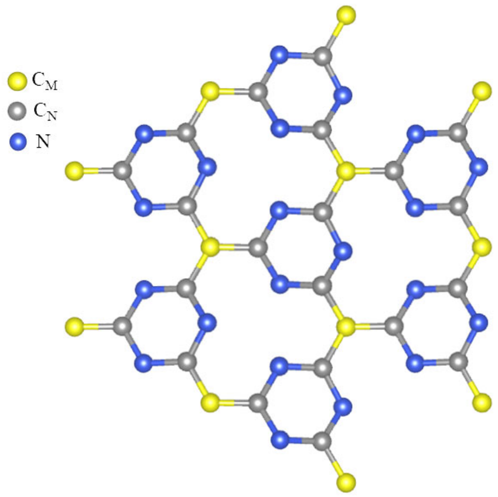

Figure S1. Atomic structure of C_4N_3 monolayer, yellow spheres are C atoms C_M with magnetic moments, silver spheres are C atoms C_N without magnetic moments, blue spheres are N atoms.

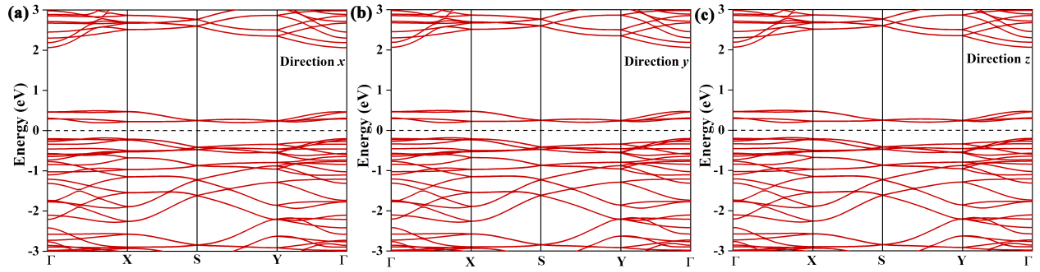

Figure S2. The spin-resolved band structures of the Pca21 C_4N_3 monolayer with spin orientations along (a) x, (b) y, and (c) z directions.

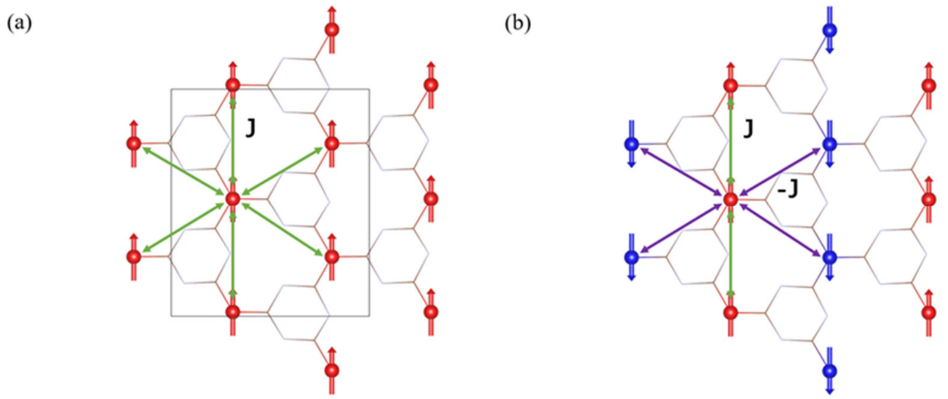

Figure S3. Schematic diagram of (a) ferromagnetic and (b) co-linear antiferromagnetic order.

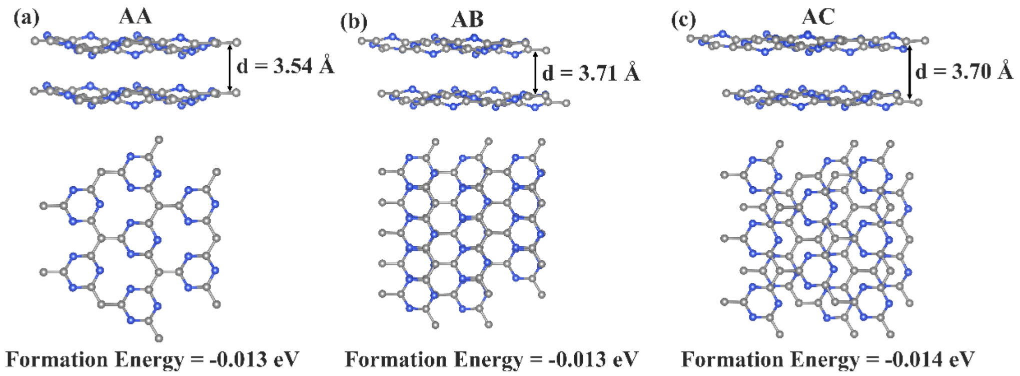

Figure S4. The side (upper) and top (lower) views for (a) AA, (b) AB, and (c) AC stacking partterns for Pca21 C_4N_3 bilayer. AA stacking: the C and N atoms of the upper layer recombined with the C and N atoms of the lower layer, respectively; AB stacking: the C and N atoms of the upper layer are overlapped with the N and C atoms of the lower layer, i.e., the AB stack is shifted by one C-C bond distance with respect to the AA stack; AC stacking: the C and N atoms of the upper layer are coincident with those of the lower layer, i.e., the AC stack is shifted by one C-N bond distance relative to the AB stack.

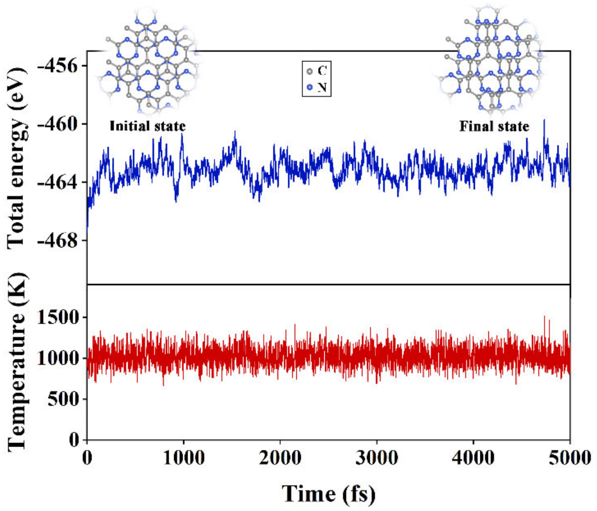

Figure S5. The AIMD simulations of the 2×2 C_4N_3 bilayer at 1000 K show the total energy (upper) and temperature (lower) fluctuations with a time-step of 1 fs for 5 ps.

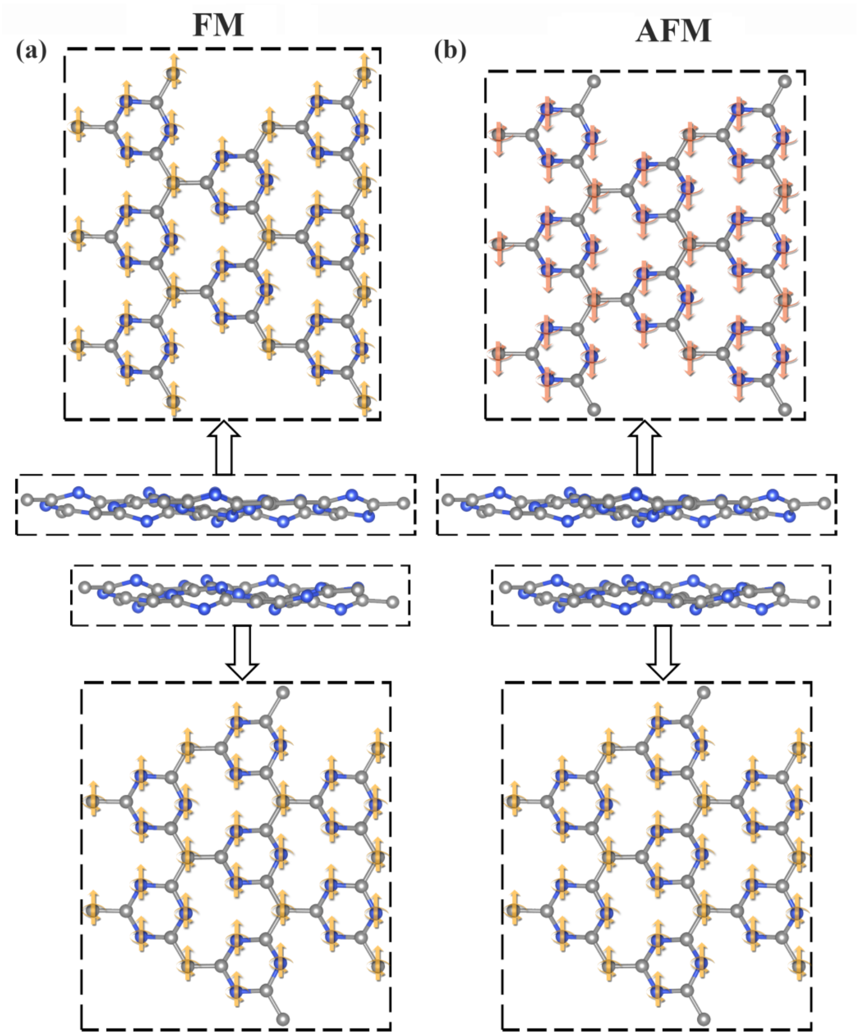

Figure S6. Schematic diagram of (a) ferromagnetic and (b) antiferromagnetic coupling of Pca21 C_4N_3 bilayer.

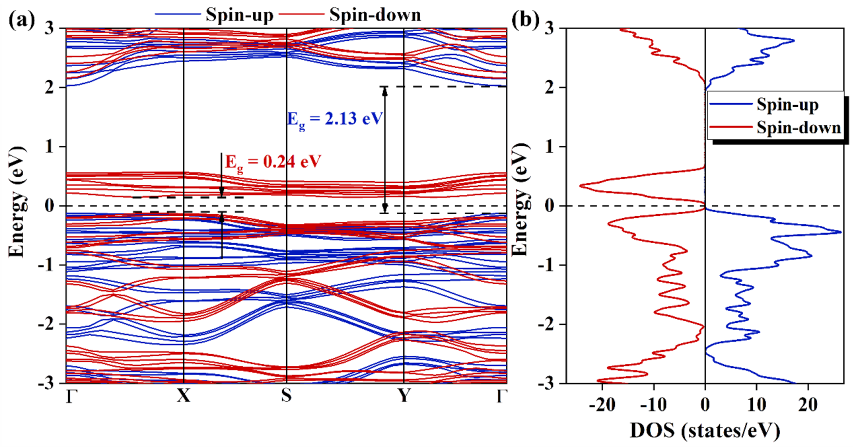

Figure S7. (a) The spin-resolved band structure of the Pca21 C_4N_3 bilayer. The spin-down and spin-up bands are marked in red and blue, respectively. The Fermi level, indicated by the black dashed line, is set to 0 eV. (b) The spin-resolved TDOS plots of the Pca21 C_4N_3 bilayer show that the TDOS in the spin-up channel are represented by blue lines, while those in the spin-down channel are indicated by red lines.

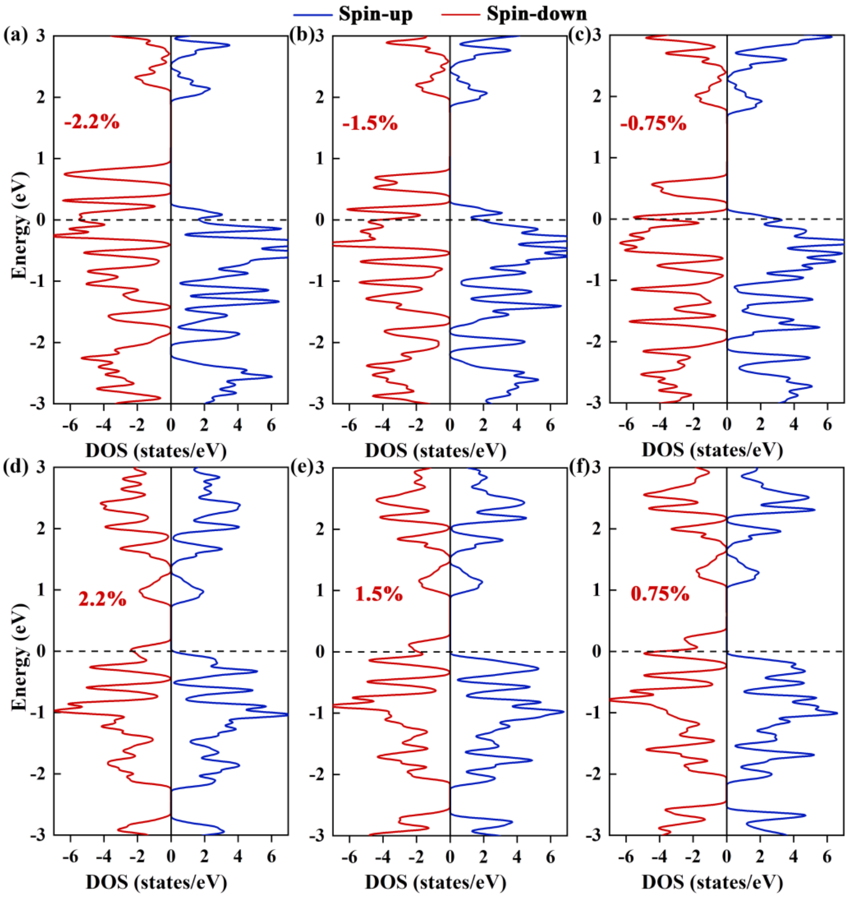

Figure S8. DOS plots of the $CO@C_4N_3$ system with the hole injection ratio of (a) 2.2%, (b) 1.5%, and (c) 0.75%, and the electron injection ratio of (d) 2.2%%, (e) 1.5%, and (f) 0.75%. The negative value of the electron injection rate means positive value of the hole injection rate.
